# Quality assured spatial dataset of wildfire containment firelines and engagement outcomes 2017 to 2024

**DOI:** 10.1038/s41597-025-05208-0

**Published:** 2025-05-28

**Authors:** Alexander Arkowitz, Scott M. Ritter, Matthew P. Thompson, Jesse D. Young, Brad Pietruszka, David E. Calkin

**Affiliations:** 1 Department of Forest and Rangeland Stewardship, Colorado State University, Fort Collins, CO 80523 USA; 2 USDA Forest Service, Rocky Mountain Research Station, 240 W Prospect Rd, Fort Collins, CO 80526 USA; 3 Pyrologix, Vibrant Planet, 111 N. Higgins Avenue, Suite 404, Missoula, MT 59802 USA; 4 USDA Forest Service, Rocky Mountain Research Station, 800 East Beckwith, Missoula, MT 59801 USA

**Keywords:** Natural hazards, Fire ecology

## Abstract

The escalation of wildfires in the USA, coupled with rising firefighting costs and decreasing workforce capacity, underscores the critical need to evaluate the efficiency and effectiveness of containment measures. However, the existing spatial data that records the locations and types of containment measures and wildfire perimeters contains numerous errors and redundancies. This paper presents a comprehensive fireline Quality Assurance and Quality Control dataset developed from the wildland firefighting operations data reported in the National Interagency Fire Center National Incident Feature Service. This improved dataset contains reliable spatial locations for fireline built during suppression operations, the associated verified fire perimeters, and identifies where containment was success or failure for fires greater than 1000 acres from 2017–2024. The improved final dataset represents critical information that was previously unavailable for assessing the success of fireline operations and incident management resource-use efficiency. The lessons learned from analyses utilizing this dataset are critical for improving the efficiency and effectiveness of the United States wildfire management system.

## Background & Summary

Wildfires in the USA have been escalating in scale and severity, which has driven up associated firefighting costs and highlighted the acute importance of assessing the effectiveness of containment measures^1–6^. Moreover, decreasing workforce capacity and systemic strain present significant challenges to wildfire management efforts. The wildfire-response system faces elevated risks, resource scarcity, critical shortages, and workforce fatigue, rendering it less capable of effectively protecting landscapes and communities^7^. Against the backdrop of resource scarcity and a strained fire management system, firefighting costs and negative wildfire impacts continue to escalate, suggesting an immediate need to understand the effectiveness of our suppression efforts. This understanding will enable the system to allocate resources more optimally and the better ensure the safety of firefighters and the protection of natural and cultural resources. The Omnibus Public Land Management Act of 2018 outlined some needed steps to achieve this by mandating the tracking of suppression operations and the analysis of wildfire suppression efficiency and effectiveness^8^, however the data currently being produced under this directive has not been fully leveraged.

Despite the strong need for data of wildfire suppression operations and federal mandates to collect and report such data, the overall quality of the available data is impacted by inconsistencies in collection methods and inaccuracies in recorded information. These deficiencies significantly impede our ability to accurately assess the success of firefighting efforts. Further assessment is gravely needed as the studies that do exist have shown that the effectiveness of retardant drops from air tankers can be as low as 43% and that only about 33% of completed fire containment lines, or firelines, are successful in holding the fire perimeter^9,10^. While common containment tactics, such as constructing and leveraging firelines, can be effective under suitable conditions^11^ they are costly, risky for firefighters during construction, and have long-term ecological consequences^12^. Therefore, the development of a consistent dataset of fireline construction is crucial for evaluating suppression and containment actions during wildfire containment efforts and characterizing the conditions under which suppression actions are likely to be successful.

Comprehensive spatial data on fireline operations and fireline construction was not available until 2017 when the National Interagency Fire Center (NIFC) began to geospatially record operational data during wildfire incidents. Over the intervening years, this data has been leveraged by scientists and geospatial fire analysts, however it contains many errors and inconsistencies that hinder its utilization. Therefore, improved data quality is necessary to understand both the interacting factors that lead to individual containment failure events as well as the drivers of incident-level fire suppression efficacy. To help address the challenges associated with using this public dataset, this paper presents a methodology for assessing the quality of fireline data and applies this method to the currently available data. This work represents a collaborative effort between researchers and geospatial wildfire analysts from the United States Department of Agriculture, Forest Service (USDA FS), and Colorado State University. The resulting dataset is the NIFC Fireline Quality Assurance and Quality Control (QAQC) dataset which includes firelines that were built prior to or coincident with the arrival of the fire and includes a number of different fireline types including hand lines, dozer lines, roads as lines, mixed construction lines, fuel breaks, burnouts, and plow lines. The dataset additionally overlays the final QAQC fireline dataset with fire perimeters allowing for evaluations into the efficacy of containment strategies.

The robust QAQC fireline dataset presented here is essential for bolstering the resilience of the currently overburdened wildfire response system. It provides the foundation for evaluating suppression effectiveness, identifying knowledge gaps, and driving improvements in incident-level decision-making and wildfire response tactics and strategies. By ensuring consistency in data collection and reporting, improving data integration for informed decision-making, fostering research opportunities, and promoting accountability and collaboration, we can enhance our understanding and management of wildfire suppression efforts.

## Methods

We processed all firelines associated with wildfire perimeters greater than 1,000 acres from the NIFC perimeter datasets from 2017–2024 to ensure the analysis focused on significant wildfire events. While the majority of these firelines were processed for inside the Continental Unites States, the dataset also includes incidents from Alaska and Hawaii. We obtained data to develop our QAQC fireline dataset from the NIFC open data site^13^. This includes historical fireline operations and wildfire perimeters. The methods and code outlined below can be downloaded from https://github.com/aarkow/NIFC-QAQC-FLE for use as a custom geoprocessing tool in ArcGIS Pro. The workflow steps for the acquisition, quality control, and determination of containment success are outlined in Table 1 and are detailed in the following sections.

**Table 1:** Workflow for Filtering and Processing GIS Firelines and Perimeters.

Step	Data	Description	Action
1	NIFC Operations: Line	Data Acquisition	Download firelines from NIFC Operational Data archives 2017–2024 as polyline features.
2	NIFC Operations: Line	Filter	Attribute query on “FeatureCat” field for: Completed Burnout, Completed Dozer Line, Completed Fuel Break, Completed Hand Line, Completed Mixed Construction Line, Completed Plow Line, Completed Road as line.
3	NIFC Operations: Line	Filter	Attribute query on “DeleteThis” field for features labeled “Yes”.
4	NIFC WFIGS and WFM RD&A Perimeters	Data Acquisition	Download wildfire perimeters from NIFS, using the WFM RD&A perimeter dataset for incidents from 2017–2019 and WFIGS perimeters from 2020–2024.
5	NIFC Perimeters	Removing Duplicates	Conducted attribution analysis to compare perimeters with identical geometry using “Find Identical” tool in ArcGIS Pro. Attribution for duplicate perimeters selected by comparing with overlayed operations data.
6	NIFC Operations: Line	Data Cleaning	Removed leading and trailing blank spaces in IRWIN ID and Incident Name fields and added missing “{}” brackets to IRWIN ID field.
7	NIFC Perimeters and Line Operations Data	Attributing Firelines to Wildfire Perimeters	Using the IRWIN ID and Incident Name fields from perimeters, lines were attributed to specific incidents. For lines missing attribution, lines were assigned to wildfires within 5 km of perimeter. If other fires were present, analysts relied on other attribution such as upload date and geometry to manually attribute line to perimeter.
8	NIFC Perimeters	Handling Complexes and Neighboring Incidents	Dissolved perimeters of complexes into one perimeter, using incident management reports to confirm overlapping burn dates using Fire Discovery and Fire Containment fields. A new field was generated to identify dissolved perimeters.
9	NIFC Operations: Line	Visual Inspection	Manually removed erroneous lines (e.g., straight lines spanning long distances over varying topography), splitting lines at last vertex before suspect geometry to retain implemented portions of line.
10	NIFC Operations: Line	Removal Of Duplicates	Used “Delete Identical” tool to remove duplicate features based on geometry and attribution, acknowledging remaining duplicates are due to slight difference in vertices or human error.
11	NIFC Perimeters and Line Operations Data	Fireline Engagement Outcome	Buffered fire perimeters by 50 meters (50 meters within and outside of perimeter). Conducted overlay analysis to classify line status (held, burned over, and not engaged).

### Fireline polylines

**Fig. 1:**
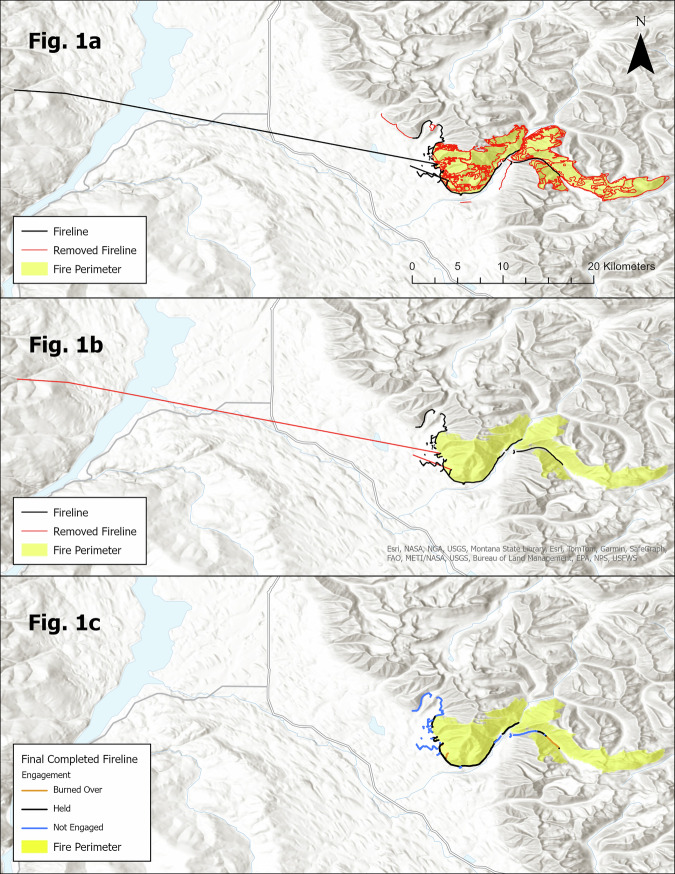
Progression of QAQC Methods on the 2017 Gibralter Ridge Fire. (**a**) Highlights the filtering of non-fireline features filtered from the Gibralter Ridge fire. (**b**) Provides an example of fireline that span great distances without vertices that were therefor removed, identified from visual QAQC. (**c**) Shows the final fireline engagement output which was created by overlaying the final fireline features with the perimeter.

First, we acquired firelines from the NIFC operations data archives as polyline features. The collection and management of these data was conducted in the field by incident Geographic Information System (GIS) analysts assigned to each wildfire incident. The digitized firelines in the raw dataset were attributed with fireline type in the “*FeatureCat” field* (otherwise referenced as the Feature Category field) which contained details related to the construction methods and suppression tactics associated with each feature. In order to differentiate firelines constructed prior to or concurrent with the fire’s arrival from activities that were conducted after the fire’s arrival we used a combination of attribute queries. To retain only firelines implemented with on-ground actions, we selected only feature with a “FeatureCat” of “*Completed Burnout*”, “*Completed Dozer Line”, “Completed Fuel Break”, “Completed Hand Line”, “Completed Mixed Construction Line”, “Completed Plow Line”, and “Completed Road as Line”*. Lines attributed in the Feature Category field as “C*ompleted Line*” and “*Line Break Completed*” were excluded as they are commonly attributed to mop up activities completed after fire growth significantly slowed or ceased^10^. Additionally, features labeled as “*Yes*” in the “*DeleteThis*” field were removed, as they were meant to be deleted by the supervising geospatial fire analyst. A visual example of this filtering method is provided by the Gibralter Ridge fire (Fig. 1a), where the red line was removed because it represented features constructed coincidently with fire arrival such as mop-up activities, or may have represented non-fireline features such as reported fire edge, proposed fireline, retardant drops, and access routes.

Though the source data also included a field called “FeatureStatus” which had categories of “Null”, “Approved”, “Archive”, “In Review”, and “Proposed”, we did not use this field to remove features as operational data specialists communicated that many firelines recorded as “Proposed” were not updated following on the ground implementation. In addition to the feedback from operational data specialists the decision to ignore “FeatureStatus” is further supported by the fact that if “FeatureStatus” Proposed lines were filtered out, it would remove 16% of firelines that were labeled “Completed…” in the Feature Category field. Finally, each fireline was attributed to the appropriate wildfire using the ‘IRWINID’ and ‘IncidentName’ fields. Of the 1,574,250 fireline features, approximately 17% of all firelines were missing linking identification information. In these instances, firelines were assigned to potential wildfires if they fell within 5 km of a wildfire’s final perimeter. These attributions were then assessed further during subsequent quality assurance and quality control steps to ensure correct fire attribution.

### Wildfire polygons

We obtained wildfire perimeters from the NIFS data archives^14,15^ from 2017 to 2024 as polygon features. These were delineated using aerial infrared imagery, GPS units, digitization, or satellite imagery. For 2017 to 2019, we used the perimeter dataset from the Wildland Fire Management Research, Development, and Application (WFM RD&A) program due to its comprehensive coverage. The WFM RD&A perimeter dataset^14^ consolidates authoritative perimeters from various agencies including the USDA Forest Service, US Department of Interior Bureau of Land Management, Bureau of Indian Affairs, Fish and Wildlife Service, National Park Service, the Alaska Interagency Fire Center, CalFire, and the Wildland Fire Interagency Geospatial Service (WFIGS). The WFM RD&A data supplement the WFIGS dataset for earlier years not covered by WFIGS. For 2020 to 2024, we used the NIFC WFIGS perimeters as they were deemed the most reliable source for high-resolution final wildland fire perimeters across the United States^15^, though currently only available back to 2020. The datasets did include some duplicated wildfire perimeters which were removed thorough attribution analysis that compared perimeter attributes with those of overlapping operations data. We used the “Find Identical” tool in ArcGIS Pro^16^ to identify perimeters that shared the same geometry but had different attribution.

Other wildfire perimeter datasets were considered, but none were used. We found fire perimeters from Monitoring Trends in Burn Severity (MTBS)^17^ to be unreliable as large, unburned islands were frequently included in the final fire perimeters which would result in firelines that successfully contained the fire being marked as failed^12^. Additionally, MTBS burn boundaries are delineated from interpretation of coarse raster Landsat or Sentinel-2 satellite imagery that is composed of cells, and not defined XY coordinates, allowing for a margin of error which could influence precise perimeter mapping. Our logic in selecting NIFS perimeters follows that of Gannon *et al*.^10^ who found these perimeters best represent the final fire extents. NIFS WFIGS and WFM RD&A perimeters were mapped using aerial infrared imagery or mixed methods that include manual adjustment to perimeters to reflect subsequent fire growth. Additionally, the firelines and NIFS perimeters draw from the same primary incident-level data, allowing for equivalent mapping resolution and accuracy^10^.

### Fireline quality assurance and quality control

We introduced quality assurance and quality control steps to account for formatting mistakes or missing fireline attributions. Many of the firelines had incorrectly formatted or missing IRWIN identification codes which we took several steps to rectify as these codes indicate the specific wildfire that was associated with the fireline (Step 7). If the IRWIN identification code or Incident Name fields contained blank spaces at the beginning or end of the string, the blanks were deleted. Furthermore, if an IRWIN identifier was missing brackets, the brackets were added. For firelines with a missing IRWIN identifier, we used a spatial buffer to overwrite missing or incorrect attributes with those reflected by the appropriate incident. This process involved creating a 5-kilometer buffer around each fire perimeter to identify and update the attributes of firelines in close proximity to the incident. We adopted this approach due to the prevalence of data quality issues, with more than 17% of all firelines containing blank or erroneous fire information, including spelling errors, invalid text, or inaccurately attributed names or IRWIN IDs. For complete transparency of this process, we implemented a spatial overlay to record and flag cases where our QAQC process overwrote existing attribution data. During this process the “Query Comparison” field was conditionally populated to account for various scenarios using the “OldName” and “OldIRWIN” fields, leading to the following attributions: *“Has Matching IRWIN and Name”, “Has Matching IRWIN ID. Name Taken From Matching IRWIN”, “Has Matching Inc Name in Same State. IRWIN Taken From Matching Name”, “Within Buffer but Both Inc Name and IRWIN Null. Both Changed to Match Perimeter”, “Within Buffer but IRWIN Null. Both Changed to Match Perimeter”, “Within Buffer but Inc Name Null. Both Changed to Match Perimeter”*, and *“Within Buffer but Both Inc Name and IRWIN Didn’t Match. Both Changed to Match Perimeter”*. It is important to acknowledge a limitation to this method, which necessitates manual intervention to rectify incorrectly overwritten firelines based on information provided in the “Query Comparison” and “Old” fields. These issues commonly arise in cases of incorrect attribution at the operational level, or from overlapping or adjacent fires. This manual verification or removal of wrongly overwritten firelines was conducted by GIS fire analysts on a per-fire basis for all fires, where each decision was scrutinized and verified by another analyst^18^.

We then applied additional steps to attribute firelines to a wildfire complex. A complex is defined as two or more individual incidents located in the same general area that were jointly managed by a single or unified command team. We found NIFS wildfire perimeters often lacked attributes identifying fire complexes. To properly attribute firelines to incidents, fires that merged or had nearby boundaries were often dissolved into one perimeter. We visually identified these perimeters and confirmed overlapping burn dates using the Fire Discovery and Fire Containment date fields. If firelines could not be properly distinguished based on attributes or matching geometry, the perimeters were dissolved into one multipart perimeter feature to represent a complex. The fire identification information from the largest fire of the complex was used. We would recommend using incident management reports and figures to attribute lines to specific perimeters of a complex if necessary.

In addition to missing and erroneous attribution, we detected numerous geometry errors. These were corrected by conducting thorough visual inspection, through which we manually removed erroneous fireline features that exhibited suspect geometry and attribution. Although this process addressed the most obvious digitization errors, some inaccuracies may still remain. One example where visual inspection resulted in the removal of a fireline was on the 2017 Gibralter Ridge fire (Fig. 1b), where we identified and removed straight lines that spanned long distances in areas where firelines could not realistically be placed. A common source of these errors occurs in ArcGIS software when users inadvertently extend a line by double-clicking the screen to exit line editing mode, rather than properly concluding the editing session. To correct these errors, we split the lines at the last vertex before the straight portion, retaining the valid section of the line while removing the problematic geometry.

Finally, we removed line features that were exact duplicates of another. We used the Delete Identical tool in ArcGIS Pro 3.2^16^ to delete these features based on both identical geometry and attribute fields, removing more than 600,000 features from our final dataset. Despite our efforts, several duplicates seemingly still exist; these lines may contain one or more differing vertices, resulting in their retention and inability to be removed using this workflow. Duplicate features may exist to represent the number of passes, different stages of line construction, or have been left in as human error. Since we could not distinguish unique causes for duplicate lines, they were removed to reduce storage space and improve processing time.

### Fireline performance

Following the development of the QAQC fireline dataset, we analyzed the outcomes of each fireline based on the final fire perimeters. Utilizing an overlay analysis, we assigned fireline status as either “burned over”, “held”, or “not engaged” within an “Engagement” field. To make this determination, we converted the fire perimeter polygons into polyline features and then expanded them by a 50-meter buffer. Any segment of a fireline intersecting this buffer zone was classified as “held”. Segments of firelines outside this buffer but within the fire’s boundaries were classified as “burned over” and segments outside both the buffer and the fire perimeter were categorized as “not engaged”.

**Fig. 2:**
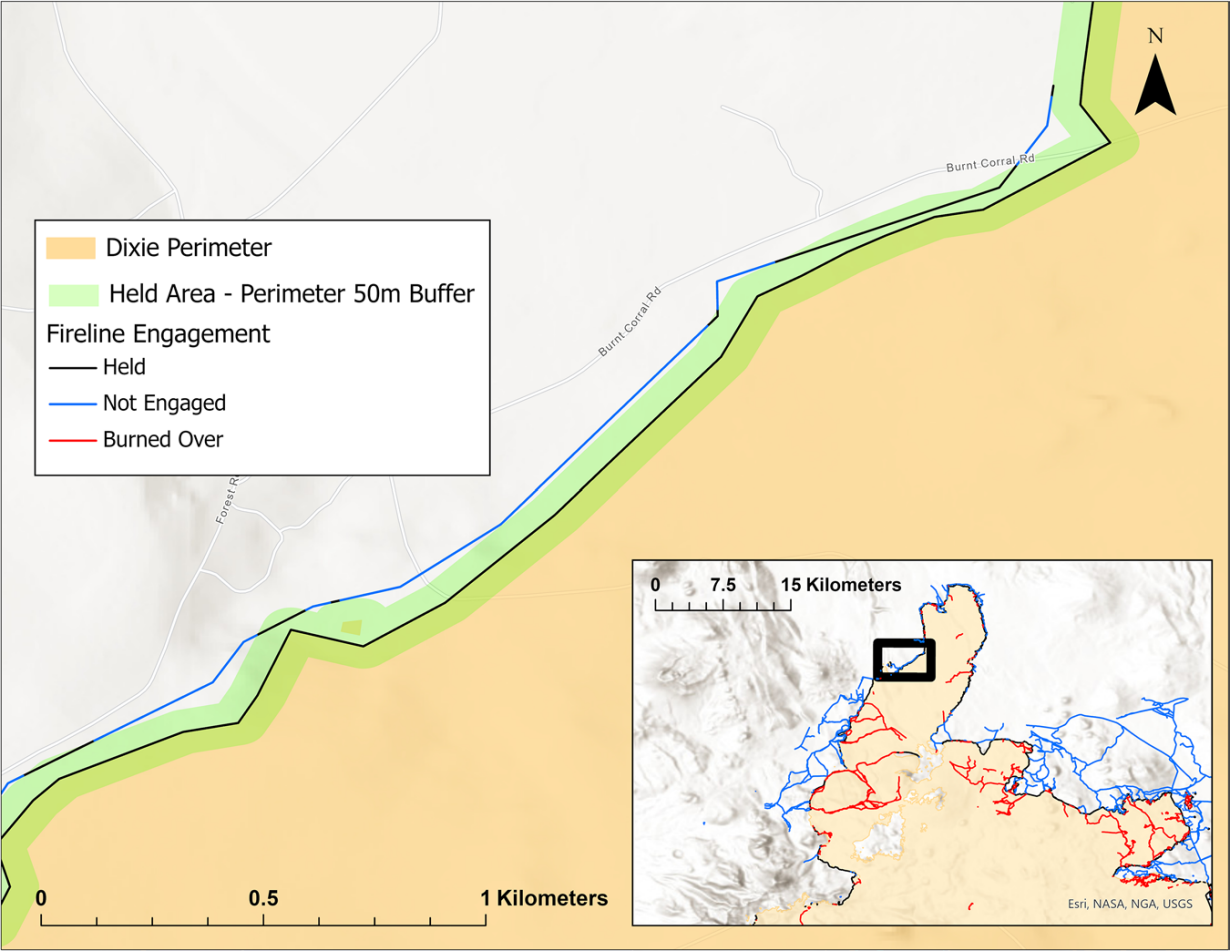
Example of 50-meter Perimeter Buffer Overlayed with Successful Held Line (Black) and Predominantly Not Engaged Secondary Backing line (Blue) on the 2021 California Dixie fire.

The inclusion of a 50-meter buffer was essential for accurately delineating the held line, given the potential spatial discrepancies in both the perimeter and fireline locations^10,12^. This buffer width was selected following an assessment of buffer sizes ranging from 25 to 100 meters. Opting for a 50-meter buffer facilitated a more precise depiction of fire behavior along the firelines^10^, as smaller buffers tended to underestimate fireline engagement and burn over, while wider buffers resulted in an overestimation of fire engagement by encompassing non-engaged, secondary, or contingency firelines, as seen in Fig. 2.

## Data Records

The QAQC fireline dataset and associated geospatial data are publicly accessible at Forest Service Research Data Archive^18^. The data is stored in a geodatabase named Fireline_Perimeter_Engagement.gdb, which includes two primary feature classes: Firelines_Engagement_2017_2024 and NIFC_Perimeters_2017_2024. These feature classes are essential for analyzing fireline engagement and the effectiveness of wildfire containment strategies across Alaska and the Continental United States from 2017 to 2024. We confirmed that all source datasets used are publicly available and verified that our reuse, modification, and redistribution fully comply with the original data providers’ licensing terms and conditions.

Fireline_Perimeter_Engagement.gdb: This ArcGIS Pro geodatabase contains spatial data specifically curated for analyzing fireline effectiveness across the United States. The geodatabase structure is straightforward, containing the following key feature classes:

Polyline Feature Class: Firelines_Engagement_17_24 Description: This feature class includes firelines constructed during wildfire suppression efforts from 2017 to 2024. Each polyline is attributed with detailed information on the type of fireline (e.g., Completed Burnout, Completed Dozer Line, Completed Hand Line), IRWIN ID, incident name, and the engagement outcome (categorized as burned over, held, or not engaged). This data supports comprehensive spatial analyses of fireline effectiveness, allowing researchers to evaluate the success of various containment strategies under different conditions.

Polygon Feature Class: NIFC_Perimeters_17_24 Description: This feature class includes wildfire perimeters from 2017 to 2024, representing spatial boundaries of fires during their active periods. Attributes include IRWIN ID, incident name, fire discovery dates, and containment dates. This feature class is used to associate fireline features with specific wildfire incidents and assess the spatial relationship between firelines and fire perimeters, providing insights into how firelines interact with and influence wildfire progression.

Both feature classes are compatible with standard GIS software (e.g., ArcGIS Pro, QGIS) and are designed to facilitate comprehensive spatial analyses. Metadata accompanying the geodatabase provides detailed descriptions of the attribute fields, data sources, and data collection protocols, supporting transparency and reproducibility in data usage.

Table 2 provides a summary of the number of incidents that included fireline construction each year, along with the total acreage impacted by these incidents. The table offers a comprehensive overview of the dataset, highlighting the scale of wildfire activity and fireline implementation across the study period. It serves as a valuable reference for understanding the annual variation in fireline usage and the extent of wildfire perimeters included in the dataset.

**Table 2:** Incidents with Firelines Per Year with Total Acreage.

Year	Incident Count	Sum of Acreage
2017	32	954,704
2018	138	4,786,202
2019	67	1,323,730
2020	203	8,153,454
2021	166	5,642,508
2022	158	3,157,606
2023	129	1,186,088
2024	258	6,621,941
**Total**	**1151**	**31,826,233**

**Fig. 3:**
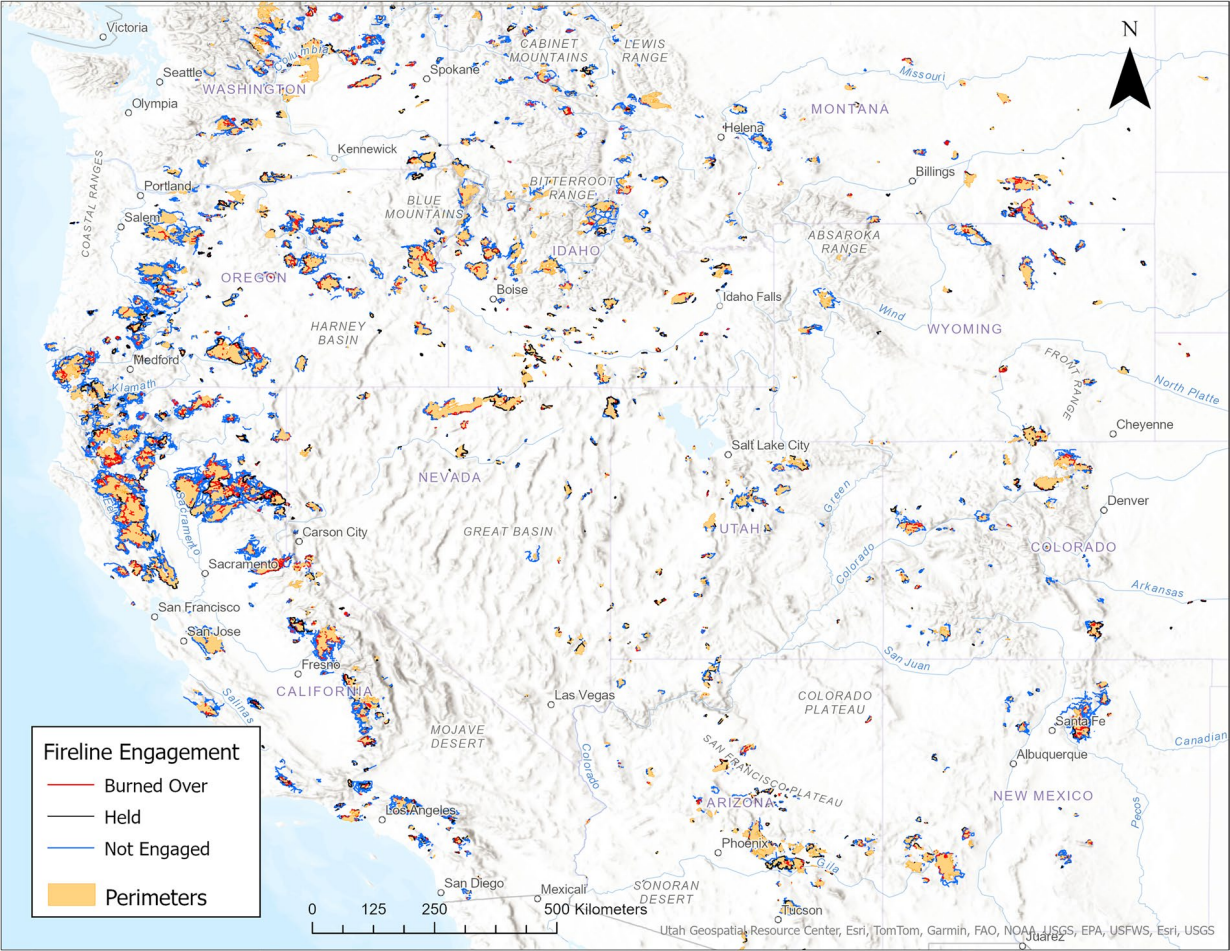
Firelines and Perimeters Across Western United States from 2017–2024.

Figure 3 provides a visual representation of the spatial distribution of firelines and wildfire perimeters across the western U.S. included in the dataset. This figure illustrates the geographic extent of the dataset and highlights the widespread application of fireline construction as a wildfire containment strategy. The map emphasizes the diversity of landscapes and fire conditions where firelines were implemented, offering context for interpreting the spatial patterns of fireline effectiveness and their role in wildfire management.

**Fig. 4:**
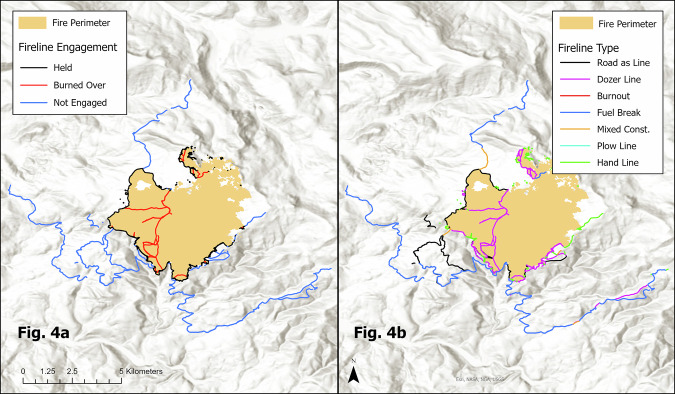
Fireline Engagement and Construction Methods During the 2022 Crockets Knob Wildfire. (**a**) Illustrates Fireline Engagement While (**b**) shows construction type.

Figure 4(a) depicts the spatial relationship between firelines and the wildfire perimeter, categorizing fireline segments into three classes: “burned over,” “held,” and “not engaged.” This classification provides critical insights into the effectiveness of fireline containment strategies, highlighting areas where firelines were breached, held successfully, or were not engaged by the fire.

Figure 4(b) categorizes firelines based on their construction methods, including “Completed Burnout,” “Completed Dozer Line,” “Completed Fuel Break,” and others. This categorization allows for an analysis of the effectiveness, cost, resource requirements, risk exposure, and ecological impact of different construction techniques in containing wildfires. The spatial representation of fireline engagement and construction methods in Fig. 3 underscores the importance of improved data collection practices, emphasizing the need for more detailed documentation of fireline construction efforts to enhance the quality and utility of future datasets, driving improvements in data collection protocols.

## Technical Validation

The QAQC dataset underwent a comprehensive validation process to ensure the accuracy and reliability of the fireline and wildfire perimeter data. The validation process encompassed several critical steps, starting with data cleaning and standardization. Initially, formatting inconsistencies in the fireline data were corrected to ensure uniformity across the dataset, including the standardization of attribute names and values. Missing attributes were addressed by cross-referencing multiple data sources and employing spatial analysis techniques to infer missing values. For example, firelines without an associated “IncidentName” were linked to the nearest wildfire perimeter within a 5 km radius. This overlay analysis was performed to assess the spatial accuracy of the fireline locations and their corresponding engagement status, with discrepancies flagged and corrected through iterative QAQC steps.

Approximately 87% of the initial 2,205,398 features in the original NIFC event line datasets required substantial correction or removal. Most removed features consisted of duplicates, incorrectly attributed data, or non-fireline elements such as mop-up activities, retardant drops, or proposed lines. To ensure the dataset accurately represented operational reality, we collaborated closely with fire management specialists to develop the logic for identifying valid fireline features reflective of on-the-ground suppression actions. Common digitization errors, including unrealistic straight-line segments and lines traversing improbable terrain, were identified and manually corrected through detailed visual inspection by GIS fire analysts. Consistency checks and inter-rater reliability assessments were employed, with multiple analysts independently reviewing data entries.

Between 2017 and 2024, over 100 fires (roughly 9% of the 1,151 total incidents) required additional manual visual QAQC to correct geometric or attribution errors that automated tools alone could not resolve. The automated workflow—such as filtering by valid feature category, removing features marked “DeleteThis = Yes,” standardizing incident names and IRWIN IDs, and deleting exact duplicates—was critical for initial data cleaning. For instance, in the raw 2018 dataset alone, automated steps removed over 100,000 non-fireline features such as proposed lines, access routes, and uncontrolled fire edge segments. These filters also resolved over 700 malformed or inconsistent IRWIN ID entries and reduced over 600,000 duplicated geometries across the full dataset. However, persistent issues like straight-line digitization errors remained. On the 2017 Gibraltar Ridge Fire, visual QAQC removed 35.76 km of erroneous firelines—lines that were either geometrically implausible or falsely attributed. This correction improved the Held-to-Total Fireline Ratio (HTR) from 0.25 to 0.48. Without these corrections, non-engaged or misaligned features would have been wrongly categorized, inflating “not engaged” segments and underrepresenting fireline success.

Error detection and correction were essential components of the validation process. Duplicate fireline and perimeter features were identified using the “Find Identical” and “Delete Identical” tools in ArcGIS Pro, which was crucial to avoid redundancy and maintain dataset integrity. Manual verification by GIS fire analysts ensured thorough visual inspection to identify and correct any anomalies or erroneous features that automated processes might have missed.

Inter-rater reliability was ensured through consistency checks, with multiple GIS analysts independently reviewing the dataset to ensure consistency in data interpretation and attribution. Any discrepancies between analysts were resolved through consensus meetings. Through these rigorous validation steps, the QAQC dataset has been established as a reliable resource for assessing fireline effectiveness and informing wildfire management strategies.

## Data Limitations

The source data does present some limitations that limit the utility of the final fireline QAQC dataset presented here. For example, the available data lacks crucial information regarding line width or the number of passes made, commonly referred to as “effort.” For instance, a bulldozer may initially clear vegetation along a designated path, then either make additional passes over the same location to more completely remove surface fuels or work parallel to the original line to create a wider containment zone. These passes can span several dozer blade widths, significantly impacting the effectiveness of the fireline and the effort and risk exposure associated with its construction. Although a field for line width is present in the original dataset, it remains unreported approximately 80 percent of the time. This gap in data collection poses a significant challenge for researchers and analysts aiming to comprehensively understand the efforts undertaken in constructing firelines. Without a clear picture of the effort expended in building these critical containment barriers, accurately studying and evaluating containment practices is hindered.

In addition, the NIFC fireline dataset could be improved at the point of creation by addressing common attribution and digitization errors with stricter editing protocols and automated error-checking tools to reduce issues such as erroneous straight-line segments. Improved data entry guidelines for GIS analysts can further mitigate these errors. Implementing standardized terminology for fireline types and their statuses would also contribute to consistency. Additionally, the dataset currently lacks detailed attribution distinguishing direct, indirect, and mop-up activities to enhance analysis accuracy. This information would provide more clarity about the strategic and tactical decisions underlying the placement of a given line as well as the timing of the action in reference to the approaching fire. These improvements, coupled with this ongoing QAQC processes, would significantly enhance reliability and utility.

While this dataset has undergone extensive quality assurance and validation, it is important to note that not all firelines constructed during wildfire suppression efforts have been mapped. Firelines included in the dataset were derived from operational records, but some may be missing due to incomplete field mapping or reporting inconsistencies. The exact percentage of missing firelines is unknown, as we cannot quantify features that were never recorded. Users should keep this in mind when analyzing fireline engagement or containment effectiveness, particularly when comparing this dataset to independent fire reports or remote sensing data.

## Usage Notes

The development of a comprehensive fireline Quality Assurance and Quality Control (QAQC) dataset holds significant potential for advancing wildfire management practices. Though this dataset covers 2017–2024, the methodology is suitable for processing data in subsequent years, and a long-term support plan is in place. The data has a range of applications in evaluating fire management teams and the fire management system as a whole.

The Fireline_Perimeter_Engagement.gdb dataset is provided in an ArcGIS Pro geodatabase format, but it is compatible with any software that can handle feature classes within geodatabases. This includes QGIS, R libraries (e.g., sf, rgdal, raster), and Python libraries (e.g., geopandas), allowing for flexible integration into various geospatial workflows. Researchers should ensure their software supports geodatabases before using the dataset.

## Data Availability

The tools used for this study are available in a GitHub repository (https://github.com/aarkow/NIFC-QAQC-FLE) designed to aid wildfire data researchers and fire management professionals in creating fireline QAQC datasets and engagement metrics. The repository includes a suite of scripts and an accompanying ArcToolbox. These tools should be downloaded onto a local drive, after which the user can open an ArcGIS Pro 3.X project and direct ArcCatalog to the files. The ArcToolbox located in the “Tool” folder contains all necessary scripts for the analysis. The general workflow begins with data preparation, where the first tool is used to ensure the integrity and accuracy of fire perimeter data. This involves verifying that perimeters do not have duplicates with different IRWIN identification numbers or incident names. After verification, the fire perimeter is exported as a single feature class for use as an input in subsequent tools. Next, the fireline QAQC tool is employed, which requires inputting a single NIFC wildfire perimeter and selecting the relevant calendar year to run the tool. A visual QAQC is then conducted to remove incorrectly attributed firelines or geometric anomalies. If necessary, a simplification step can be applied using tool 2B to create a simplified dissolved fireline dataset. Engagement analysis is then performed using tool 3, which attributes engagement statuses to QAQCed firelines, labeling them as “Held,” “Not Engaged,” or “Burned Over”. This tool also creates a buffered output for overlay analysis. There are some limitations to consider. The tools require ArcGIS Pro version 3 or higher and may not be compatible with earlier versions. Proper formatting and naming of the file directory are essential, as the directory structure and file naming conventions must meet the toolkit’s requirements for smooth operation. Additionally, a stable internet connection is necessary for tools that download data from online sources or require data syncing. Connectivity interruptions could result in incomplete data processing or other errors. For more detailed information on the tools and their use, users are encouraged to refer to the tool descriptions and the embedded comments in the Python code itself.
